# Incidence disparities of obstructive sleep apnea-associated lung cancer by gender; Korean National Health Insurance data analysis

**DOI:** 10.3389/fonc.2023.1214279

**Published:** 2023-07-19

**Authors:** Marn Joon Park, Kyung-Do Han, Jae Hoon Cho, Ji Ho Choi

**Affiliations:** ^1^ Department of Otorhinolaryngology-Head and Neck Surgery, Inha University Hospital, School of Medicine, Inha University, Incheon, Republic of Korea; ^2^ Department of Statistics and Actuarial Science, Soongsil University, Seoul, Republic of Korea; ^3^ Department of Otorhinolaryngology-Head and Neck Surgery, School of Medicine, Konkuk University, Seoul, Republic of Korea; ^4^ Department of Otorhinolaryngology-Head and Neck Surgery, Bucheon Hospital, College of Medicine, Soonchunhyang University, Bucheon, Republic of Korea

**Keywords:** lung cancer, sleep apnea, obstructive sleep apnea, incidence, national health programs

## Abstract

**Introduction:**

Obstructive sleep apnea (OSA) is known to increase the risk of various cancers. By analyzing the Korea National Health Insurance Service (KNHIS) registry, the impact of OSA on the lung cancer incidence was analyzed in a retrospective cohort group.

**Methods:**

A retrospective cohort of adult patients newly registered with OSA in the KNHIS data from 2007 to 2017 was included and observed until December 2019 (12 years). The main outcome measure was newly diagnosed lung cancer. The control group was set with age and sex that matched those in the OSA group.

**Results:**

The hazard ratio (HR) of OSA for lung cancer incidence showed a significantly reduced HR of 0.87 (95% CI, 0.82–0.93). The observed significance of this finding was limited to male OSA patients [HR, 0.84 (95% CI, 0.78–0.90)], while no significant association was found in female OSA patients [HR, 1.05 (95% CI, 0.91–1.21)], irrespective of their age.

**Discussion:**

OSA patients have a lower risk of developing lung cancer, but this risk reduction is gender-specific, as female OSA patients do not show a reduction in hazard ratio.

## Introduction

The incidence of obstructive sleep apnea (OSA) is increasing worldwide, affecting approximately 15% of males and 5% of females in the North American population (1). In South Korea, the prevalence of OSA is reportedly 4.5% in males and 3.2% in females (2). Patients with OSA show intermittent hypoxemia (IH) with or without hypercapnia, resulting in functional changes in the autonomous nerve system (3–5), as well as structural or molecular changes in the cardiovascular (6), neurologic (7), immune (8), or endocrine organs (9). Therefore, OSA acts as an independent predisposing factor for developing various cerebrovascular and metabolic disorders (10). One additional aspect of managing OSA lies in its efficacy in reducing the risk of the development of, for example, ischemic heart disease, stroke, and type 2 diabetes mellitus (DM) with decreased morbidity (11, 12).

Interestingly, it has been claimed that OSA may be positively associated with various malignant neoplasms in humans (13). Justeau et al. (14) reported that patients with more severe nocturnal IH who were untreated showed a significant increased risk in all-cancer incidence independently; of all the cancers, lung cancer showed the most statistical significance.

Lung cancer stands as the leading cause of cancer-related deaths in men and second-greatest cause in women; approximately 2.1 million patients were diagnosed, and 1.8 million deaths were recorded worldwide in 2018 (15). In South Korea, the crude incidence of lung cancer reported in 2019 was 58.4 per 100,000, affecting males and females at rates of 79.4% and 37.4%, respectively (16). Although the exact mechanism through which OSA develops into lung cancer is uncertain, the possible carcinogenetic and cancer-progression potential of OSA in lung cancer was reported in the previous study (17).

To date, numerous meta-analysis studies regarding OSA and cancer incidence have been published, suggesting that lung cancer is one of the cancers correlated with OSA (12, 17–20). Nevertheless, a study focusing on the incidence of newly diagnosed lung cancer in patients diagnosed with OSA in a large, nation-wide cohort has not yet been published to the authors’ knowledge, particularly in the East Asian population. Additionally, no previous studies have conducted a subgroup analysis on the impact of gender differences regarding OSA and lung cancer development.

The authors sought to determine the incidence and hazard ratio of lung cancer in patients with OSA using a large, nationwide retrospective cohort stretching twelve years, utilizing data from the Korea National Health Insurance Service (KNHIS) database. The study incorporated analyses of subgroups based on gender and age.

## Materials and methods

### Ethical declaration

This study was approved by the Institutional Review Board (IRB) of Inha University Hospital (IRB no. 2022-11-004). The IRB has reviewed and approved the study design and issued an exemption for the informed consent of the study subjects. The authors adhered precisely to the research standards while being formally monitored by the IRB.

### Source of data: the KNHIS database

The current study was conducted with data from the KNHIS. Since 2000, the South Korean government has mandated that all South Korean citizens are registered and covered by the KNHIS in terms of seeking medical care (21). Upon submitting a formal dissertation protocol in addition to ethical approval from the official review committee, the KNHIS offers the usage of the data registered in the KNHIS archive. Each person registered in the KNHIS receives a unique resident registration number, thereby eliminating the possibility of duplication or omission upon data analysis. Both inpatient and outpatient claims are examined by the KNHIS, with data on the patients’ demographics, clinical diagnoses, medical expenses, and interventions for diagnostic or therapeutic purposes. The KNHIS reviews all medical claims and classifies and stratifies the received data based on the Korean Standard Classification of Diseases, 6th edition (KCD-6), a modified version of the International Classification of Diseases, 10th Revision (ICD-10).

### Acquisition of the data from the KNHIS dataset

The identification of patients diagnosed with OSA was performed with a search of the operational code for OSA (G47.30) in the KNHIS dataset. The patients’ demographic data for age, gender, and income level were obtained. The details of the patients’ medical history, including diagnoses of hypertension (HTN), diabetes, dyslipidemia, ischemic heart disease, chronic obstructive pulmonary disease, and stroke, were additionally gathered from the claimed insurance data. The operational definition and search requirements regarding each disease are further elaborated in **Table 1**.

**Table 1:** The KNHIS database search criteria and processes for patients with each condition.

Name of Each Disease		Search Protocols of Each Disease
Study subjects	OSA	Patients who were registered with G47.3 in the ICD-10 code in the KNHIS dataset, with a minimum of a single claim
Primary endpoint measurements	Lung cancer	Patients registered with C33 or C34 in the ICD-10 code in the National Medical Expenses Support Program, with a minimum of a single registration
Various diseases analyzed for confounding variables	Diabetes	Patients who were prescribed anti-diabetic medication under ICD-10 code E11-14, with a minimum of a single prescription per year, in the KNHIS dataset
	Hypertension	Patients who were prescribed anti-hypertensive medication under ICD-10 code I10, I11, I13, or I15, with a minimum of a single prescription per year in the KNHIS dataset
	Dyslipidemia	Patients who were prescribed anti-hypertensive medication under ICD-10 code E78, with a minimum of a single prescription per year in the KNHIS dataset
	Stroke	Patients who were registered with I63 or I64 in the ICD-10 code in the KNHIS dataset, with a minimum of a single claim
	COPD	Patients who were registered with G47.3 in the ICD-10 code in the KNHIS dataset, with a minimum of a single claim
	IHD	Patients who were registered with G47.3 in the ICD-10 code in the KNHIS dataset, with a minimum of a single claim

KNHIS, Korea National Health Insurance Service; OSA, obstructive sleep apnea; ICD, International Classification of Diseases; COPD, chronic obstructive pulmonary disease; IHD, ischemic heart disease.

In the KNHIS dataset, each individual’s diagnosis is represented by a unique designation (e.g., G4730 for OSA, C33 or C34 for lung cancer, E11 to 14 for diabetes, etc.). Therefore, we could only acquire the presence of specific disease diagnoses and the date of each diagnosis. As stated previously, in order to search for OSA patients during the research period, we searched the KNHIS database for individuals who were claimed with the G4730 diagnosis code during the study period, as well as other diseases.

### Primary endpoint and study design

This study included adults (age ≥ 20) who were newly diagnosed with OSA (G47.30) from Jan 2007 to Dec 2017. The primary endpoint of this observational study was the incidence of newly diagnosed lung cancer in these newly diagnosed OSA patients. Patients who were newly diagnosed with OSA in that period were enrolled as a retrospective cohort, and the claimed insurance data of the cohort were retrospectively reviewed up to the end of 2019, creating an observation period of twelve consecutive years.

The presence of newly diagnosed lung cancer (ICD-10 code C 33 or C 34) was re-viewed in this cohort period in a retrospective manner in the National Medical Expenses Support Program registry (**Table 1**). The patients were excluded according to the timing of lung cancer diagnosis or if they had been withdrawn from the KNHIS upon death. The time interval between the OSA diagnosis and lung cancer diagnosis was calculated; it was defined as a ‘person-year at risk’ for developing a new onset of lung cancer, which was calculated for all included subjects.

To compare the cumulative risk of lung cancer incidence in OSA patients, a control group was recruited. The controls were chosen using propensity score matching by gender and age and selected from among patients without an OSA diagnosis from Jan 2007 to Dec 2017. The overall number of participants in the control group was set to be five times that of the OSA patients.

**Figure 1:**
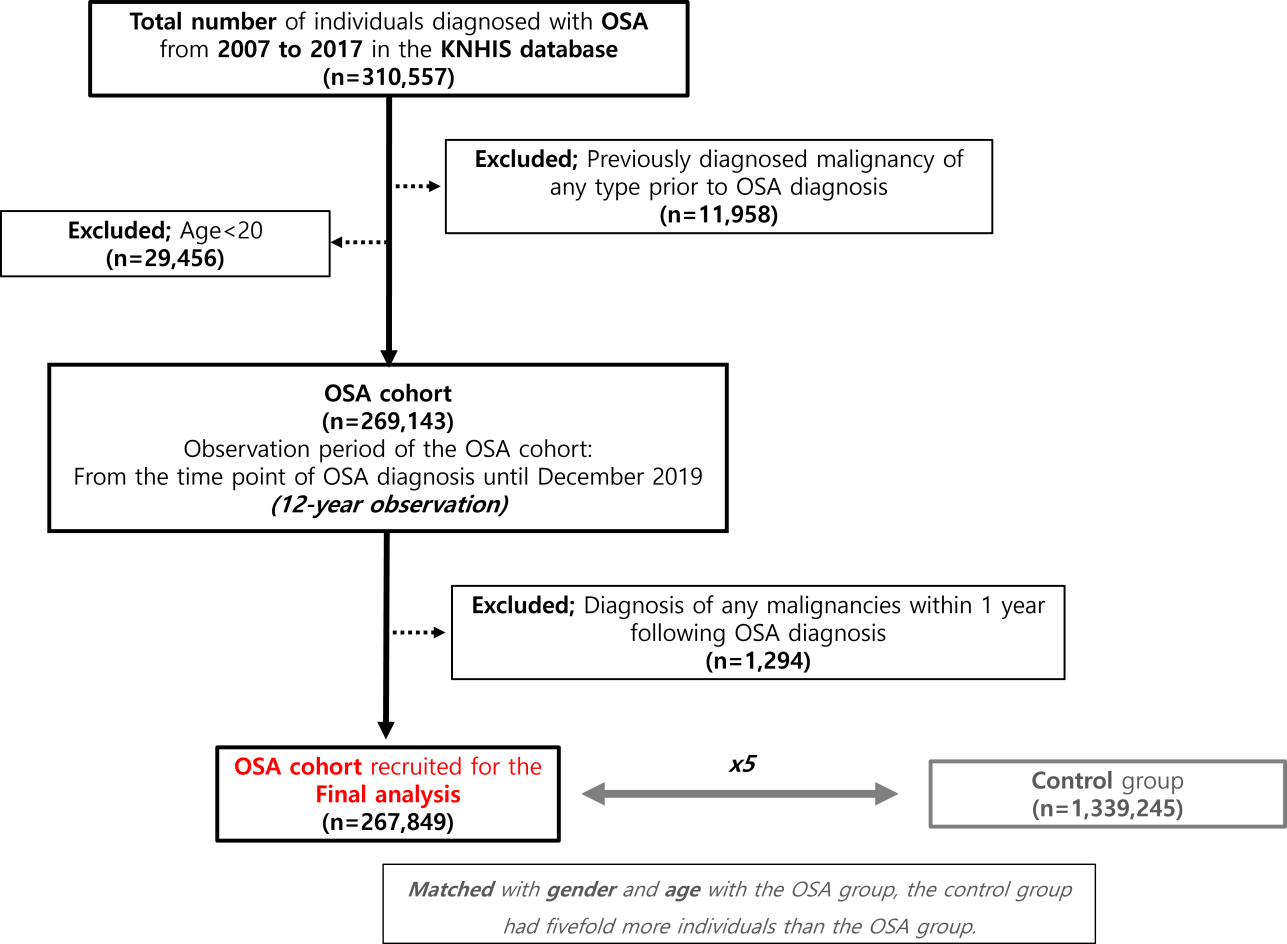
Recruitment of patients with obstructive sleep apnea (OSA) and the control group. A flow diagram elaborating the recruitment process of the OSA group and control group from the Korea National Health Insurance Service (KNHIS) database.

Prior to the enrollment, patients with any malignant tumors (determined through a search for all operation codes for malignant neoplasm) were excluded from both the OSA and control groups. A flowchart regarding the details of the retrospective OSA cohort with the selection process of the control groups is further illustrated in **Figure 1**.

### Statistical analysis

Descriptive statistical analysis was conducted for various types of demographic and clinical information. Regarding each type of clinical variable, Student’s t-test or a chi-square test was adopted to compare the OSA and control groups. To compare the incidence rate of newly diagnosed lung cancer patients between the OSA group and control group, a log-rank test was conducted, and a cumulative-incidence plot was drawn. The hazard ratio (HR) for lung cancer development was calculated with the adoption of two different Cox proportional hazards models in both the OSA and control groups. In model A, the covariate was not considered for HR calculation. In model B, the HR was adjusted for all cofounding variables: income level, HTN, diabetes, dyslipidemia, stroke, COPD, and ischemic heart disease. Additionally, subgroup analyses were performed to deter-mine the odds ratio (OR) of OSA for developing lung cancer in the various subgroups, i.e., gender (two groups) and age (three groups), using a logistic regression analysis.

Two hypotheses underlie the Cox proportional hazards model. First, stratum survival curves must have proportionate hazard functions over time. Residual plots indicate the linearity of the log hazard-covariate relationship. During the retrospective cohort research, Korean lung cancer incidence increased somewhat, which may have impacted our findings. Logistic regression models analyze the association between a binary result and a collection of covariables. Logarithmic regression analysis might be biased if the sample size is too small. We mitigated this bias by using a large sample size in our analyses. The p value for the interaction was calculated to validate the statistical reliability of the sub-group analyses. A p-value that was less than.05 was considered statistically significant. All statistical analyses were executed in a two-tailed manner, and the results were presented with a 95% confidence interval (CI). SAS ver. 9.4 (SAS Institute Inc., Cary, NC, USA) or R ver. 3.2.3 (The R Foundation for Statistical Computing, Vienna, Austria) was utilized for statistical analyses.

## Results

According to the KNHIS records, the total number of registered individuals in 2007 was 49,570,064. Throughout the patient recruitment phase ranging from January 2007 to December 2017, a total of 310,557 patients were registered with newly diagnosed OSA, as depicted in **Figure 1**. The control group comprised a total of 1,339,245 individuals who were recruited. During the observation period spanning from January 2007 to December 2019, the retrospective cohort’s mean follow-up time interval was 5.9 ± 3.1 years, as determined by the calculated mean and standard deviation. A p-value below.05 was deemed statistically significant.

### Demographic differences between the OSA and control groups

Various demographic data and clinical diagnoses of the OSA group and control group are presented in **Table 2**. As the OSA patients’ gender and age were matched upon the recruitment of the control group, the age and sex between the two groups showed statistical consistency, with a p-value of 1.0. By contrast, other demographical variables showed significantly different distributions between the two groups. The OSA group had a significantly higher rate of underlying comorbidities, including chronic obstructive pulmonary disease (COPD). The OSA group had a significantly lower number of subjects on the lowest incomes (**Table 2**).

**Table 2:** Demographics of OSA patients and controls.

	OSA (*n* = 267,849)	Controls (*n* = 1,339,245)	p-Value
Age (years)	45.68 ± 13.17	45.68 ± 13.17	1.000
Age ≥ 65 years	22220 (8.3)	111100 (8.3)	1.000
Gender (male)	203026 (75.8)	1015130 (75.8)	1.000
No. of subjects with income in the bottom quintile	35208 (13.14)	238775 (17.83)	<0.001
Diabetes	18622 (6.95)	81748 (6.1)	<0.001
Hypertension	66391 (24.79)	210889 (15.75)	<0.001
Dyslipidemia	47290 (17.66)	131316 (9.81)	<0.001
Stroke	3176 (1.19)	4367 (0.33)	<0.001
COPD	21428 (8)	59753 (4.46)	<0.001
IHD	2564 (0.96)	5815 (0.43)	<0.001
Follow-up duration (years)	5.92 ± 3.14	5.89 ± 3.13	<.0001

Values presented in mean ± standard deviation or number (%).

OSA, obstructive sleep apnea; COPD, chronic obstructive pulmonary disease; IHD, ischemic heart disease.

### The impact of OSA on lung cancer development

**Figure 2:**
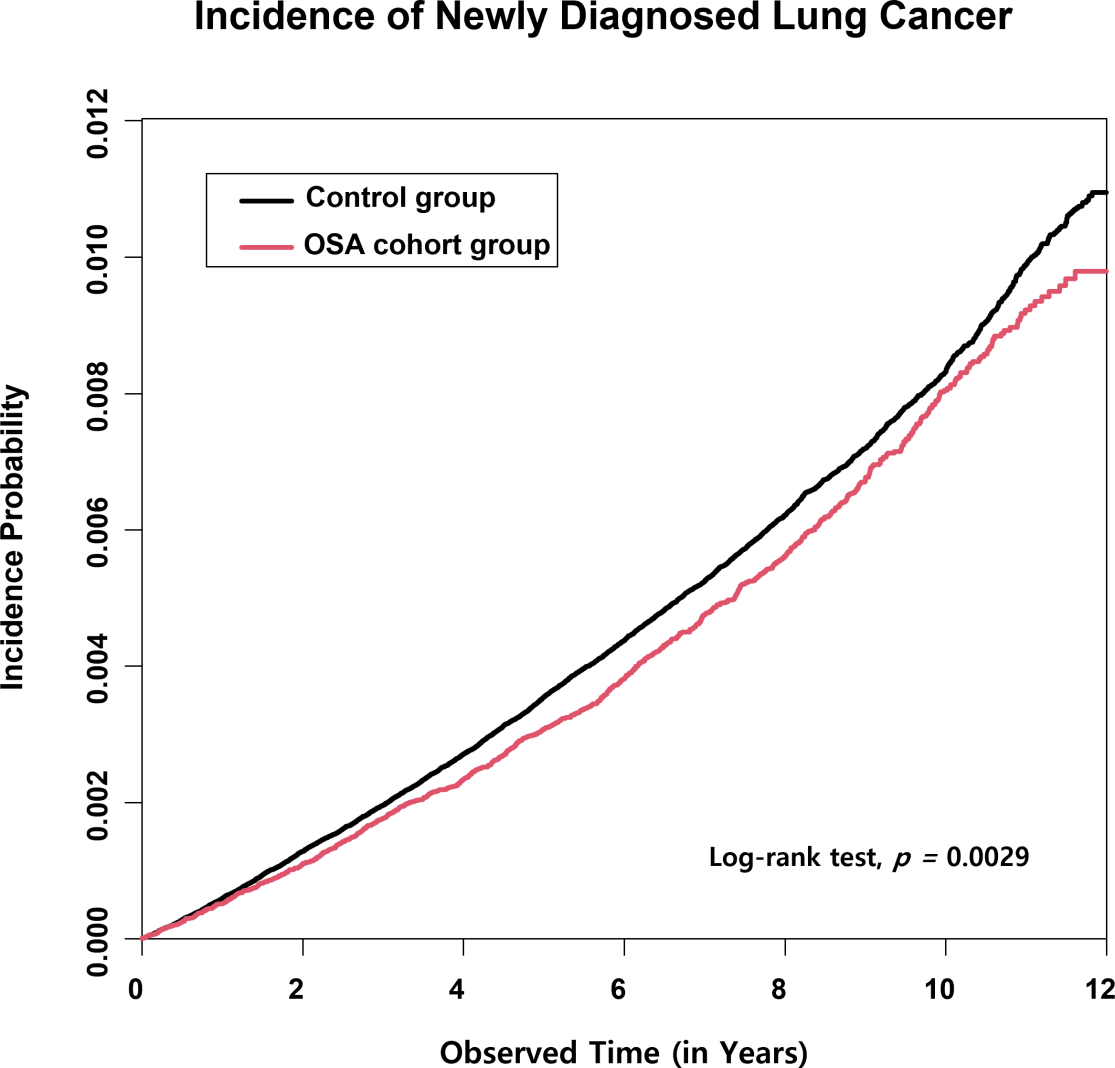
Lung cancer cumulative incidence plot in the obstructive sleep apnea (OSA) group and the control group. The cumulative incidence of lung cancer in a twelve-consecutive-year retrospective cohort showed a statistically significantly reduced risk of lung cancer development in the OSA group compared with the control group.

In the 12-year retrospective cohort, the OSA group showed a significantly reduced rate of cumulative lung cancer incidence than the control group. As represented in the cumulative incidence plot for the development of new onset lung cancer, a significant difference in the incidence probability between the OSA and control groups was observed (p=0.003) (**Figure 2**).

The mean follow-up duration (from the point of OSA diagnosis to the lung cancer diagnosis, censoring, or termination of the cohort) of the OSA patients was 5.92 ± 3.14 years, whereas the follow-up duration of the control group was 5.89 ± 3.13 years (p<0.001; **Table 2**). The significantly shorter observation time interval observed in the control group than in the OSA group indicates that OSA patients were lesser prone to developing lung cancers than the control group.

The HR calculated from the Cox proportional hazard model to evaluate the impact of OSA on the development of lung cancer showed a statistically significantly reduced risk of developing lung cancer in the non-adjusted model (Model A, HR of 0.91 (95% CI, 0.85–0.97)) and a greater reduction in the adjusted model [Model B, HR of 0.87 (95% CI, 0.82–0.93)] (**Table 3**).

**Table 3:** The HR of OSA for developing lung cancer.

	*n*	Event (Newly diagnosed lung cancer)	Rate (%) (Event/*n*)*100	HR Calculated in Model A^1^	HR Calculated in Model B^2^
				HR (95% CI)	HR (95% CI)
Control	1339245	6141	0.779	1 (ref)	1 (ref)
OSA	267849	1122	0.708	0.91 (0.85–0.97)	0.87 (0.82–0.93)

HR, hazard ratio; OSA, obstructive sleep apnea; CI, confidence interval.

^1^Model A was derived using Cox proportional hazards analysis with no confounding variables.

^2^Model B was derived using Cox proportional hazards analysis adjusted for sex, age, subjects’ income levels, diabetes, hypertension, dyslipidemia, stroke, chronic obstructive pulmonary disease, and ischemic heart disease.

In both the gender and the three different age subgroups, the male subgroup showed only a significantly reduced adjusted HR of 0.84 (95% CI, 0.78–0.90; **Table 4**). By contrast, this significantly reduced effect of OSA on lung cancer development did not prevail in the female subgroup, as the adjusted HR was 1.05 (95% CI, 0.91–1.21). By contrast, no significant risk reduction or increase were shown in the young adult (aged between 20 to 40) and middle-aged adult (aged between 40 to 65) (**Table 5**). On the other hand, the old age sub-group (aged > 65) showed a decreased HR of 0.74 and 0.70 in both models (**Table 5**). The p values for interactions in all subgroup analyses were less than 0.025, indicating the statis-tical validity of the subgroup analysis.

**Table 4:** The HR of OSA with lung cancer development according to each gender group.

	Individuals who were diagnosed with lung cancer following OSA diagnosis					
		N	Event (Newly diagnosed lung cancer)	Rate (%) (Event/*n*)*100	HR Calculated in Model A^1^	HR Calculated in Model B^2^
					HR (95% CI)	HR (95% CI)
Male	Control	1,015,130	5094	0.849	1 (ref)	1 (ref)
	OSA	203,026	897	0.742	0.87 (0.81–0.94)	0.84 (0.78–0.90)
Female	Control	324,115	1,047	0.555	1 (ref)	1 (ref)
	OSA	64,823	225	0.597	1.08 (0.93–1.24)	1.05 (0.91–1.21)
P for interaction					0.011	0.007

HR, hazard ratio; OSA, obstructive sleep apnea; CI, confidence interval.

^1^The HR in Model A was derived using Cox proportional hazards analysis with no confounding.

^2^The HR in Model B was derived using Cox proportional hazards analysis adjusted for sex, age, subjects’ income levels, diabetes, hypertension, dyslipidemia, stroke, chronic obstructive pulmonary disease, and ischemic heart disease.

**Table 5:** The HR of OSA with lung cancer development according to each age group.

	Individuals who were diagnosed with lung cancer following OSA diagnosis					
		N	Event (Newly diagnosed lung cancer)	Rate (%) (Event/*n*)*100	HR Calculated in Model A^1^	HR Calculated in Model B^2^
					HR (95% CI)	HR (95% CI)
Age 20-40 Years	Control	474515	181	0.063	1 (ref)	1 (ref)
	OSA	94903	45	0.078	1.25 (0.90–1.73)	1.23 (0.89–1.71)
Age 40-65 Years	Control	753630	3843	0.863	1 (ref)	1 (ref)
	OSA	150726	756	0.844	0.98 (0.90–1.06)	0.95 (0.88–1.03)
Age > 65 Years	Control	111100	2117	3.828	1 (ref)	1 (ref)
	OSA	22220	321	2.849	0.74 (0.66–0.83)	0.70 (0.63–0.79)
P value for the interaction					< 0.001	< 0.001

HR, hazard ratio; OSA, obstructive sleep apnea; CI, confidence interval.

^1^The HR in Model A was derived using Cox proportional hazards analysis with no confounding.

^2^The HR in Model B was derived using Cox proportional hazards analysis adjusted for sex, age, subjects’ income level, diabetes, hypertension, dyslipidemia, stroke, chronic obstructive pulmonary disease, and ischemic heart disease.

A further breakdown of the analysis was performed to evaluate the gender-specific effects according to different age groups (**Supplementary Table 1**). Regardless of male and female sex, there was no significance in the lung cancer risk according to the presence of OSA diagnosis. The demographics of the male OSA and female OSA patients exhibited some distinctive differences regarding the development of lung cancer (**Supplementary Table 2**). The male OSA lung cancer group showed significantly higher rates of older age, low income, and all comorbid systemic diseases than the male OSA lung cancer-free patients. On the other hand, the female OSA lung cancer patients showed only an increased number of older age, hypertension, and COPD patients than the female OSA lung cancer-free patients.

## Discussion

The results of this current study, which were obtained from the nation-wide retrospective cohort from 2007 to 2017 with a 12-year observation period (2007 to 2019), showed that the adult patients diagnosed with OSA had a significantly reduced risk of developing lung cancer. This reduced risk was significantly valid when the cofounding demographic variables were adjusted. However, in the subgroup analysis according to each gender, only the male OSA patients showed a significantly reduced adjusted HR of 0.84 (95% CI; 0.78–0.90), whereas the adjusted HR of the females did not show such statis-tical significance. Despite potential limitations such as selection bias and inadequate adjustment for various lung cancer risk factors, our analysis of the KHNIS dataset suggests that male patients with obstructive sleep apnea have a lower risk of developing lung cancer. Gender disparity in the carcinogenesis pathway of lung cancer may exist, as females may not exhibit the same pattern.

The strength of our study lies in its large statistical power, owing to the fact that all South Korean citizens are mandatorily covered by the National Health Insurance Service. In addition, the observation of both the OSA and the control group for twelve consecutive years in a retrospective manner may strongly support the causal association of the carcinogenic potential of OSA with lung cancer in our results when compared with cross-sectional or case-control studies. Moreover, the application of a subgroup analysis of the lung cancer risk of OSA according to the gender and age-group differences in the large retrospective cohort in this study was not reported in previous studies. To the authors’ knowledge, this study provides the largest recorded power and causality for revealing the incidence of lung malignancies in OSA patients.

Recently, sleep-related breathing disorders (SBD), including OSA, as independent risk factors for developing various malignant tumors in the human body, have gained interest from many researchers (13, 14, 22). The chronic IH in OSA followed by various responses in the neuroendocrine, cardiovascular, and respiratory organs may facilitate tumor growth and progression in various organs (23). Many studies from various nations have suggested OSA as an independent risk factor for all-cancer incidence. For instance, the relative risk was 1.26 in Sillah et al.’s study (24), and three meta-analyses resulted in all-cancer-risk values of 1.49, 1.53, and 1.52. Additionally, previous results showed dose–response relationships between OSA and cancer incidence. Palamaner Subash Shantha et al. (18) reported that overall cancer incidence and mortality were higher by up to three times in severe OSA than in mild-to-moderate OSA. However, some studies presented results that opposed this finding, suggesting instead that OSA was not a significant risk factor in the development of all types of cancer (25, 26). Some cancers are more likely to be correlated with OSA, while other types of malignant tumors are not (14, 20). In the previous analyses, in terms of cancers of various organs, colorectal (27), breast (28), prostate (9), pancreas (29), and renal-cell carcinomas (29) were shown to be significantly affected by OSA, as OSA served as a significant risk factor for developing these malignancies. Together, the outcomes of previous clinical and epidemiological studies suggest the potential clinical impact of OSA on the incidences of various cancers.

Contradictory results were obtained regarding whether OSA may affect lung cancer incidence and mortality in previous studies. In some cross-sectional studies with relatively large numbers of subjects, the incidence of SBD or OSA reached 49% to 80% in all patients newly diagnosed with lung cancer, and approximately half of the lung cancer patients had moderate-to-severe OSA (30–33). Huang et al. (32) reported that stage III and IV advanced-lung cancer patients with an apnea–hypopnea index (AHI) greater than 30 had a three-year mortality rate of 80%, which was reduced to 25% in patients with an AHI less than 15. This study strongly connected the effect of the IH of OSA with carcinogenesis and tumor progression in lung cancer. A large-scale cohort followed for a further 5 years and population-based studies further support OSA as an independent risk factor for higher lung cancer incidence. Huang et al. (32) reported an HR of 1.52 (95% CI, 1.07–2.16), and Jara et al. (34) reported an HR of 1.32 (95% CI, 1.27–1.37), showing a significantly in-creased HR of OSA in lung cancer incidence. Nonetheless, Kendzerska et al. (35) reported an HR of 1.38 (95% CI, 0.94–2.03) showing no statistical significance; insignificance was also reported in Gozal et al.’s study (29). Furthermore, Sillah et al. (24) reported that OSA was associated with a significantly reduced lung cancer incidence, as their HR value was 0.66 (95% CI, 0.54-0.79). The disagreement in the previous studies suggests the need for a well-designed, large, nation-wide cohort study with a relatively long duration of observation. The 12-year retrospective nation-wide cohort in our study design might explain and further support the discordant results on whether OSA might play a role in lung cancer development.

Our results demonstrate that the HR for developing new-onset lung cancer in OSA patients compared with the control group with different ages, sexes, incomes, and various comorbid diseases was 0.91 (95% CI; 0.85–0.97), showing a significantly reduced risk of developing lung cancer. An even greater risk reduction was observed in a model in which cofounding variables were considered, showing a significantly adjusted HR of 0.87 (95% CI; 0.82–0.93). Moreover, the cumulative incidence plot showed a significant difference between the OSA and control groups in the twelve-year retrospective cohort. The results indicate that OSA may not be associated with lung cancer development in the general adult population, regardless of other cofounding clinical variables. Our results provide a remarkable addition to the knowledge on this subject, as previous researchers disagreed over whether OSA increases the risk of lung cancer development.

In our study, there was a significant reduced risk of lung cancer in OSA individuals. Like our results, Sillah et al. s’ report where the OSA showed as a significantly protective agent for developing lung cancer (24). In Siallah et al. s’ study, all the included subjects were aged over 64 years. Similarly, our study subgroup analysis showed a significantly reduced risk only in the senile age (age>65) group, which carefully suggests a rather protective effect of OSA for lung cancer development in the geriatric population, but not in the young and mid-aged adult population. Moreover, Christensen et al. reported an increased risk of lung cancer in OSA patients in the patients under 50 years old (25), and an animal study on an intermittent hypoxic model revealed rather a protective effect for lung cancer development in the aged group, which the younger group did not (36). The impact of age on the increased susceptibility to OSA in lung cancer development should be more clarified and discovered in the future studies.

Theoretically, the sleep fragmentation (SF) and IH observed in OSA patients are two major features that could explain the increased cancer incidence in the OSA population. Although the exact mechanism of IH in carcinogenesis is not yet understood, the frequent hypoxia followed by normoxia in IH mimics a condition similar to the reperfusion injury in tissues that undergo ischemic stress (37). The production of reactive oxygen species by the endothelial cells of vascular structures exposed to chronic IH may predispose patients to carcinogenesis in normal tissues (38). Moreover, IH may up-regulate various hypoxia-inducible factors (HIFs) in many organs, which may promote carcinogenesis (39, 40). On the other hand, SF has been shown to result in activated sympathetic tone, chronic inflammation, and altered immune cell functions, all of which may promote carcinogenesis in various organs (41, 42). Furthermore, it has been also suggested that nasal obstruction, which is a common accompanying findings in OSA patients might also play a role attributing in chronic inflammation and oxidative stress, which all together lead to carcinogenesis (43).

Owing to the fact that OSA is caused by a narrowing of the upper airway in most cases, the lower airway tract and the lung are anatomically the principal areas affected by the sustained decrease in oxygen concentration following hypoventilation in OSA patients (44). Thus, the carcinogenic potential of IH in lower airway tract cells in OSA patients is a field of interest for many researchers (45). The chronic inflammation as a result of IH may synergize with various known inhalant carcinogens, such as tobacco smoking, in the lower airway, thus facilitating the formation of a microenvironment suited to lung cancer development (46). Another possible mechanism for lung cancer development and progression in OSA is *via* HIFs, as HIF-2α has shown potential for carcinogenesis, angiogenesis, and metastasis in lung cancer in various studies (38–40, 45, 47). Furthermore, Wnt/β-catenin signaling, which is thought to be enhanced through interaction with up-regulated HIF pathways, has been shown to activate the oncogenic potential in non-small-cell carcinomas of the lung (48).

In addition, managing OSA has been shown to reduce oxidative stress, free radicals, and inflammation, all of which can reduce the risk of carcinogenesis. Oxidative stress is an imbalance between the production of reactive oxygen species (ROS) and the ability of antioxidants to neutralize them. Multiple forms of cancer, including laryngeal cancer, have been associated with its presence (49, 50). Due to intermittent hypoxia and reoxygenation during sleep, obstructive sleep apnea syndrome (OSAS) has also been correlated with oxidative stress. OSAS may contribute to the development of laryngeal cancer by fostering chronic inflammation and oxidative stress, which can cause DNA damage and promote tumor growth (49). Moreover, OSAS may impede immune function, making it more difficult for the body to combat cancer cells.

Furthermore, it has been suggested that disruptions in the human circadian rhythm potentially lead to carcinogenesis in various organs (51); this includes lung cancer, which is one of the cancers related to disrupted circadian rhythms (52). As suggested in Koritala et al.’s article, various biomarkers and molecular changes indicating a disrupted circadian rhythm are observed in OSA patients, associated with sleep fragmentation and sleep arousal (53). Melatonin, an endogenous molecule secreted in the pineal gland and regulated by the circadian rhythm (54), is reportedly insufficiently secreted in OSA patients (55–57). Interestingly, many scholars have suggested the defensive role of melatonin in the carcinogenesis pathway (58, 59), and the decreased secretion of melatonin in OSA patients may lead to increased cancer risk (60). Taken together, the results of previous cellular and animal studies offer a molecular basis for further support for the oncogenic potential of OSA, including carcinomas of the lung.

Interestingly, significant lung cancer risk reduction was seen only in the males, and not in the females, regardless of the patients’ age group. The exclusive significance of female susceptibility to lung cancer after OSA highlights the novelty of our study. Wu et al. (20) reported an increased risk of OSA for all-cancer development, especially in the female population. However, in the previous literature, it was not clarified whether OSA increases cancer risk predominantly in females; for instance, Cheong et al. (17) raised this issue as a limitation in their meta-analysis. Previous studies, including some meta-analyses, reported a significantly increased risk of OSA with lung cancer incidence (17, 32, 34), while some studies did not (20, 29), and others even reported a significantly decreased risk (24). These contradictory results are thought to result from differences between the demo-graphic characteristics in each study. Huang et al.’s study (32) was based on a female-only cohort, which showed an HR of 1.52, while Jara et al.’s study (34) was mostly (94%) on male patients, resulting in an HR of 1.32 for lung cancer incidence in OSA patients, showing a significantly increased lung cancer incidence in both genders. However, it must be noted that both studies were derived from predominantly white and relatively homogenous occupational groups, as Huang et al.’s study (32) consisted of nurses and Jara et al.’s study (34) consisted of veterans. Thus, the conclusions derived from their results might be limited for the general population. Furthermore, it should be stated that most of the previous studies were published in Western countries, in which those of East Asian heritage form only a small proportion of the population.

The lung cancer epidemiology in East Asian populations showed a distinctive pat-tern compared with the Caucasian population; in South Korea, 36% were never-smokers, and more than up 70% of the never-smoking lung cancer patients were female and were histologically diagnosed with adenocarcinoma-expressing epidermal growth factor receptor (EGFR) mutations (61). Interestingly, Marhuenda et al. (62) reported different tumor proliferation patterns, expression levels of epithelial cell adhesion molecule, and different amounts of HIF-1α nuclear translocation between squamous cell carcinoma and EGFR mutation-positive and -negative adenocarcinomas derived from human pulmonary cell lines.

The exclusive increase in lung cancer incidence in the female population regardless of age might aid in the addition of new knowledge. Since our results showed the susceptibility of female patients with OSA specifically to developing lung cancer, the question of whether pulmonary tissues from never-smoking females are more vulnerable to IH in developing adenocarcinoma with EGFR mutations would be an interesting investigation topic in the future. An increased HR of 1.52 in Huang et al.s’ study which only consists of female (32), shows a higher HR compared with HR in Jara et al.s’ study, showing a HR of 1.32, which their study group only included male patients (34). The results of these two cohort studies goes in line with our results, suggesting an increased susceptibility to lung cancer development in the female OSA than the male OSA. Nonetheless, these two studies are separate, two independent studies, and there are no well-designed systemic study investigating on the gender difference in the incidence of lung cancer development in OSA patients. Therefore, we suggest that this gender difference in the incidence of lung cancer in OSA group might be an interesting topic in the future research.

Although our study presented some unprecedented findings with a twelve-year retrospective cohort on a nation-wide scale, we acknowledge some limitations that must be declared. First, we were not able to consider the social history, such as smoking and alcohol abuse, or familial history of lung cancer, which are some of the known major risk factors for lung cancer (63), in each patient, as these demographics were not included in the KNHIS data. Although we acquired data on the presence of COPD diagnosis, which is known to occur at a high rate in heavy tobacco smokers, we declare that these data might not quite represent the effect of tobacco smoking in our study. Furthermore, the study sub-jects’ nutritional status (i.e., body mass index), which is known to be associated with OSA, was not evaluated, as this information is not available in the KNHIS registry. Second, we were not able to provide a subgroup analysis on the subtypes of lung cancer in terms of the histological diagnoses or genetic profiles of lung cancer among the study subjects. Third, the KNHIS data only provided patients diagnosed with OSA, with no details regarding the OSA severity (represented as apnea-hypopnea index, etc.), obesity degree represented as body mass index, or whether the patients underwent treatment for OSA. Therefore, we were not able to analyze the dose-response of OSA severity and confounding impact of obesity in lung cancer development in OSA individuals. Along with the severity of OSA, BMI shall also be considered to elucidate this matter in the future study. Fourth, our research classified adult population as those over 20, whereas others have characterized it as 18 to 19. Consequently, the disparity in adult age criteria may limit the comparability of OSA’s effect on lung cancers with other studies. Last, the control group was only set to have 5 times larger number than the study group, and only matched with age and gender. These might possess the possibility of selection bias, thereby limit the results of our study. Furthermore. it should be stated that there is the possibility of an undiagnosed OSA population in the ‘control’ group in our cohort, as the OSA group in our cohort consisted of patients who received medical care and were confirmed to have OSA by a physician.

## Conclusions

Adults with OSA had a slightly lower hazard ratio (0.87) for lung cancer development in the Korean population. The risk reduction was observed only in male OSA patients in the subgroup analysis by gender. Our study is the first study in the literature to raise the gender differences in the OSA impact in lung cancer development, analyzed with the largest number of retrospect cohort of 12 years on a national-wide scale.

## Data availability statement

The raw data supporting the conclusions of this article will be made available by the authors, without undue reservation.

## Ethics statement

This study was approved by the Institutional Review Board (IRB) of Inha University Hospital (IRB no. 2022-11-004). Written informed consent for participation was not required for this study in accordance with the national legislation and the institutional requirements.

## Author contributions

Conceptualization: MP, JCho, and JChoi. Formal analysis: K-DH, JCho, and JChoi. Investigation: K-DH, JCho, and JChoi. Methodology: K-DH, JCho, and JChoi. Resources: JCho and JChoi. Writing–original draft: MP, JCho, and JChoi. Writing–reviewing and editing: MP, JCho, and JChoi. All authors contributed to the article and approved the submitted version.
