# Supplementary material for: Incidence disparities of obstructive sleep apnea-associated lung cancer by gender; Korean National Health Insurance data analysis
Source: Front Oncol. 2023 Jul 19;13:1214279. doi: 10.3389/fonc.2023.1214279 (PMC10395835; doi:10.3389/fonc.2023.1214279)
Supplement: Supplementary file 1 [file Table_1.docx]

Supplementary Material

Incidence disparities of obstructive sleep apnea (OSA)-associated lung cancer by gender; Korean National Health Insurance Data Analysis

**Marn Joon Park ^1^, Kyung-Do Han ^2^, Jae Hoon Cho ^3,*,†^, and Ji Ho Choi ^4,*,†^**

^1^ Department of Otorhinolaryngology-Head and Neck Surgery, Inha University Hospital, Inha University School of Medicine, 27, Inhang-ro, Jung-gu, Incheon 22332, Republic of Korea

^2^ Department of Statistics and Actuarial Science, Soongsil University, 369 Sangdo-ro, Dongjak-gu, Seoul 06978, Republic of Korea;

^3^ Department of Otorhinolaryngology-Head and Neck Surgery, Konkuk University School of Medicine, 120-1, Neungdong-ro, Gwangjin-gu, Seoul 05030, Republic of Korea

^4^ Department of Otorhinolaryngology-Head and Neck Surgery, Soonchunhyang University College of Medicine, Bucheon Hospital, 170, Jomaru-ro, Bucheon 14584, Republic of Korea

*** Corresponding Author: Jae Hoon Cho and Ji Ho Choi**^†^jaehoon@kuh.ac.kr (Jae Hoon Cho) and handsomemd@hanmail.net (Ji Ho Choi)
† These authors contributed equally to this work and should be considered co-corresponding authors.

# Supplementary Tables

**Supplementary Table 1. OSA patients vs controls: gender-specific age effects on lung cancer development.**

|  |  |  | **N** | **Event**  **(Newly diagnosed lung cancer)** | **Rate (%)**  **(Event/*n)*100*** | **HR Calculated in**  **Model A^1^** | **HR Calculated in**  **Model B^2^** |
| --- | --- | --- | --- | --- | --- | --- | --- |
|  |  |  |  |  |  | **HR (95% CI)** | **HR (95% CI)** |
| Age 20-40 Years | Male | Control | 395,730 | 146 | 0.061 | 1 (ref) | 1 (ref) |
|  |  | OSA | 79,146 | 37 | 0.077 | 1.27 (0.88–1.82) | 1.23 (0.86–1.78) |
|  | Female | Control | 78,785 | 35 | 0.071 | 1 (ref) | 1 (ref) |
|  |  | OSA | 15,757 | 8 | 0.081 | 1.15 (0.53–2.47) | 1.11 (0.52–2.40) |
| P for interaction | | | | | | 0.167 | 0.810 |
| Age 40-65 Years | Male | Control | 552,455 | 3,184 | 0.970 | 1 (ref) | 1 (ref) |
|  |  | OSA | 110,491 | 601 | 0.907 | 0.93 (0.86–1.02) | 0.92(0.84–1.01) |
|  | Female | Control | 201,175 | 659 | 0.564 | 1 (ref) | 1 (ref) |
|  |  | OSA | 40,235 | 155 | 0.664 | 1.18 (0.99–1.40) | 1.16 (0.97–1.38) |
| P for interaction | | | | | | 0.019 | 0.021 |
| Age > 65 Years | Male | Control | 66,945 | 1,764 | 5.371 | 1 (ref) | 1 (ref) |
|  |  | OSA | 13,389 | 259 | 3.816 | 0.71 (0.62–0.81) | 0.67 (0.59–0.77) |
|  | Female | Control | 44,155 | 353 | 1.572 | 1 (ref) | 1 (ref) |
|  |  | OSA | 8,831 | 62 | 1.384 | 0.88 (0.67–1.15) | 0.84 (0.64–1.10) |
| P for interaction | | | | | | 0.155 | 0.151 |

HR, Hazards ratio; OSA, obstructive sleep apnea; CI, confidence interval.

^1^ HR in Model A was derived using Cox proportional hazards analysis with no confounding.

^2^ HR in Model B was derived using Cox proportional hazards analysis adjusted with the sex, age, subjects’ income level, diabetes, hypertension, and dyslipidemia, stroke, chronic obstructive pulmonary disease, and ischemic heart disease.

**Supplementary Table 2. Demographics of retrospective OSA cohort according to the presence of lung cancer diagnosis.**

| **Retrospect OSA cohort (patients diagnosed with OSA from 2007 to 2017), (n=267,849)** | | | | | | | | | |
| --- | --- | --- | --- | --- | --- | --- | --- | --- | --- |
|  | ***All OSA patients (n=267,849)*** | | | ***Male OSA patients (n=203,026)*** | | | ***Female OSA patients (n=64,823)*** | | |
|  | **Lung cancer-free**  **(n=266,727)** | **Lung cancer development**  **(n=1,122)** | ***p*-Value** | **Lung cancer-free**  **(n=202,129)** | **Lung cancer development**  **(n=897)** | ***p*-Value** | **Lung cancer-free**  **(n=64,598)** | **Lung cancer development**  **(n=225)** | ***p*-Value** |
| Male gender | 202129 (75.8) | 897 (80.0) | **0.001** |  | | |  | | |
| Mean age (years) | 45.6±13.2 | 58.5±10.2 | **<.001** | 44.3±12.6 | 58.57±10.1 | **<.001** | 49.66±14 | 58.37±10.64 | **<.001** |
| Age 20-40 Years | 94858 (35.6) | 45 (4.0) | **<.001** | 79109 (39.1) | 37 (4.1) | **<.001** | 15749 (24.4) | 8 (3.6) | **<.001** |
| Age 40-65 Years | 149970 (56.2) | 756 (67.4) |  | 109890 (54.4) | 601 (67) |  | 40080 (62.1) | 155 (68.9) |  |
| Age > 65 Years | 21899 (8.2) | 321 (28.6) |  | 13130 (6.5) | 259 (28.9) |  | 8769 (13.6) | 62 (27.6) |  |
| No. of subjects with income in the bottom quintile | 35039 (13.1) | 169 (15.1) | 0.057 | 22959 (11.4) | 133 (14.8) | **0.001** | 12080 (18.7) | 36 (16.0) | 0.300 |
| Diabetes | 18448 (7.0) | 174 (15.5) | **<.001** | 13551 (6.7) | 150 (16.7) | **<.001** | 4897 (7.6) | 24 (10.7) | 0.081 |
| Hypertension | 65892 (24.7) | 499 (44.5) | **<.001** | 48372 (23.9) | 412 (45.9) | **<.001** | 17520 (27.1) | 87 (38.7) | **<.001** |
| Dyslipidemia | 46977 (17.6) | 313 (27.9) | **<.001** | 33182 (16.4) | 254 (28.3) | **<.001** | 13795 (21.4) | 59 (26.2) | 0.075 |
| Stroke | 3145 (1.2) | 31 (2.8) | **<.001** | 2231 (1.1) | 26 (2.9) | **<.001** | 914 (1.4) | 5 (2.2) | 0.307 |
| COPD | 21224 (8.0) | 204 (18.2) | **<.001** | 14701 (7.3) | 162 (18.1) | **<.001** | 6523 (10.1) | 42 (18.7) | **<.001** |
| IHD | 2543 (1.0) | 21 (1.9) | **0.002** | 1974 (1.0) | 18 (2.0) | **0.002** | 569 (0.9) | 3 (1.3) | 0.469 |

Values presented in N (%) or Mean±SD

OSA, obstructive sleep apnea; COPD, chronic obstructive pulmonary disease; IHD, ischemic heart disease.
